# Identification of risk features for complication in Gaucher’s disease patients: a machine learning analysis of the Spanish registry of Gaucher disease

**DOI:** 10.1186/s13023-020-01520-7

**Published:** 2020-09-22

**Authors:** Marcio M. Andrade-Campos, Laura López de Frutos, Jorge J. Cebolla, Irene Serrano-Gonzalo, Blanca Medrano-Engay, Mercedes Roca-Espiau, Beatriz Gomez-Barrera, Jorge Pérez-Heredia, David Iniguez, Pilar Giraldo

**Affiliations:** 1 Grupo Español de Enfermedades de Depósito Lisosomal, Sociedad Española de Hematología y Hemoterapia, (GEEDL), Zaragoza, Spain; 2 Hospital del Mar Institut Hospital del Mar d’Investigacions Mèdiques, Barcelona, Spain; 3 Fundación Española para el Estudio y Terapéutica de la Enfermedad de Gaucher y otras lisosomales (FEETEG), Zaragoza, Spain; 4 Grupo de Investigación en Enfermedades Metabólicas y Hematológicas Raras (GIIS-012), Instituto Investigación Sanitaria Aragón, Zaragoza, Spain; 5 Departamento de Bioquímica, Biología Molecular y Celular, Universidad de Zaragoza, Zaragoza, Spain; 6 Centro de Imagen. Vivo, Zaragoza, Spain; 7 Kampal Solutions, Universidad de Zaragoza, Zaragoza, Spain; 8 Instituto de Biocomputación y Física de Sistemas Complejos (BIFI), Zaragoza, Spain

**Keywords:** Gaucher disease, Machine learning, Bone crisis, Neoplasia, ERT

## Abstract

**Background:**

Since enzyme replacement therapy for Gaucher disease (MIM#230800) has become available, both awareness of and the natural history of the disease have changed. However, there remain unmet needs such as the identification of patients at risk of developing bone crisis during therapy and late complications such as cancer or parkinsonism. The Spanish Gaucher Disease Registry has worked since 1993 to compile demographic, clinical, genetic, analytical, imaging and follow-up data from more than 400 patients. The aims of this study were to discover correlations between patients’ characteristics at diagnosis and to identify risk features for the development of late complications; for this a machine learning approach involving correlation networks and decision trees analyses was applied.

**Results:**

A total of 358 patients, 340 type 1 Gaucher disease and 18 type 3 cases were selected. 18% were splenectomyzed and 39% had advanced bone disease. 81% of cases carried heterozygous genotype. 47% of them were diagnosed before the year 2000. Mean age at diagnosis and therapy were 28 and 31.5 years old (y.o.) respectively. 4% developed monoclonal gammopathy undetermined significance or Parkinson Disease, 6% cancer, and 10% died before this study. Previous splenectomy correlates with the development of skeletal complications and severe bone disease (*p* = 0.005); serum levels of IgA, delayed age at start therapy (> 9.5 y.o. since diagnosis) also correlates with severe bone disease at diagnosis and with the incidence of bone crisis during therapy. High IgG (> 1750 mg/dL) levels and age over 60 y.o. at diagnosis were found to be related with the development of cancer. When modelling the decision tree, patients with a delayed diagnosis and therapy were the most severe and with higher risk of complications.

**Conclusions:**

Our work confirms previous observations, highlights the importance of early diagnosis and therapy and identifies new risk features such as high IgA and IgG levels for long-term complications.

## Introduction

Gaucher Disease (GD)(MIM#230800; MIM#231000; MIM#230900) is the most common lysosomal storage disorder (LSD) [1, 2]; some of the most common problems for GD patients are difficulty in diagnosis [3], appearance of complications, variability in the intensity of symptoms and absence of curative treatments with decreased quality of life [4, 5]. Clinical characteristics of GD are well established, but there remains a lack of information due to the singularity of the cases [6]. Also, it has been impossible to define a complete phenotype-genotype correlation [7–9] or to create a prognosis model for complications. Because of these the initiative to create registries has been developed by different institutions, research groups, and pharmaceutical companies; allowing a continuous improvement in the knowledge of the disease [10–12].

GD has a pan-ethnic distribution with cases described worldwide. Outside the Ashkenazi Jewish population, incidence ranges from 1 in 40,000 to 1 in 100,000 inhabitants; however, in the Ashkenazi population a higher incidence has been found (1 in 2500) and GD is not considered a rare disease [13]. Three types have been described: type-1 GD (GD1; MIM#230800), or the non-neuronopathic GD form, is the most common in western countries and it is characterized by the absence of primary involvement in the central nervous system; type-2 GD (GD2; MIM#230900) is the acute neuronopathic form with very severe cases, all of them with a short lifespan of less than 2 years; and type-3 GD (GD3; MIM#231000), or the juvenile/adult neuronopathic form, described for first time in 1959 [14], is characterized by neurological affectation and also involvement of other organs such as lungs, cardiac valves, and kyphosis, among other manifestations [15–18].

The application of Enzymatic Replacement Therapy (ERT), which began in 1991, has significantly improved awareness of the disease, and has changed the characteristics and expectations of patients as well as the experience of everyone involved in GD management. Nowadays, ERT offers a secure therapy for GD patients with 3 different available enzymes worldwide, two of them in Europe (Imiglucerase, Sanofi-Genzyme and Velaglucerasa alfa, Takeda pharmaceuticals), taliglucerase alfa obtained from plant cell-expressed is until now non approval in EU [19–22]. Since 2004, Substrate Reduction Therapy (SRT) has been developed for GD treatment, first with one iminosugar (Miglustat, Actelion Pharmaceuticls) and more recently with a ceramide mimetic (Eliglustat Tartatre, Sanofi-Genzyme) [23–25] expanding the therapeutic options to GD patients. However, there is still the need to develop means of identifying the small number of patients who are at risk of bone crisis while receiving ERT, as well as those who are at risk of developing late complications such cancer or parkinsonism.

The Spanish Gaucher Disease Registry (SGDR) has worked since 1993 to compile demographic, clinical, genetic, analytical and imaging data of Spanish GD patients (currently numbering 361 GD1, 36 GD, and 21 GD3). The Registry has allowed us to calculate GD prevalence in Spain (about 1/100,000 inhabitants) and to identify the *GBA* (MIM*606463) variants distribution in the population [12, 18].

In the last decades, the explosion of all kind of data has driven to the use of different big data and machine learning techniques for many applications in the healthcare and bioinformatics fields (several reviews can be seen in references [26–28]. In particular the application of computational tools and correlations network techniques for the analysis of data can provide new insights into the relationship between different variables and with the disease, as well as informative and descriptive visualizations [28, 29]. The main objective of this project is to identify new correlations among the patient characteristics and to made a first approximation to the development of prediction models for the risk of late complications.

## Patients and methods

### Patients

Since the establishment of the SGDR coordinated by the Fundación Española para el Estudio y Terapéutica de la Enfermedad de Gaucher y otras lisosomales (FEETEG), a total of 418 GD patients have been reported in Spain. All patients included in the SGDR provided informed consent for the collection and use of the information and biological samples for research projects, all according to the Helsinki declaration of 1963 revised in October 2013, and in accordance with European Regulation 2016/679 on the protection of personal data and the free movement of such data. For this study, ethics and scientific FEETEG boards gave their approval.

All the registered patients were included except those diagnosed with GD2 and those who had less than 70% of baseline data available (Table 1). Of 418 patients in the SGDR, 358 (85.6%) were analysed.

**Table 1:** Variables

**Demographics**	
Gender	M/F
Birthdate	dd/mmm/year
Age at diagnosis	years
Cosanguinity	Y/N
Family history of PD	Y/N
Death date	Y/N
Survival	years
**Clinical Data**	
GD-DS3	mild moderate severe
Spleen removal	Y/N
Liver size	cm
Spleen size	cm
Previous bone crisis	Y/N
**Image Data**	
S-MRI	0-> 9
DEXA	Z score T score
**Analytical Data**	
Hemoglobin	g/dL
WBC	10^9^/L
Platelets	10^9/^L
B12 vitamin level -serum concentrations,	pg/mL
Iron concentration	mg/dL
Cholesterol	mg/dL
Triglycerides	mg/dL
HDL-cholesterol	mg/dL
LDL-cholesterol	mg/dL
AST/ALT	UI
GGT/ alkaline phosphatase	UI
Bilirrubin	mg/dL
IgG-, IgA-, IgM	mg/dL
**Diagnosis**	
GCase activity	nmol/mL/h
*GBA* genotype	NM_000157
**Biomarkers**	
ChT	nmol/mL/h
*CHIT1* genotype NM_0003465	Homozygous Heterozygous N
CCL18/PARC	ng/mL
GluSph	ng/mL
Ferritin	mcg/L
**Follow-up**	(5-25 y)
Age to start therapy	years
Type of therapy	ERT SRT N
New bone crisis	Y/N
Joint replacement	Y/N
Neoplasia	Y/N
PD	Y/N
Other comorbidities	Y/N

*S-MRI* Spanish magnetic resonance score, *DEXA* Bone mineral density, *GD-DS3* Severity category of GD, *WBC* white blood cell count, *GCase* glucocerebrosidase, *ChT* Chitotriosidase, *CCL18/PARC* Chemokine ligand 18/Pulmonary and activation-regulated chemokine, *GluSph* Glucosylsphyngosine, *PD* Parkinson Disease

### Study design

In collaboration with Kampal Data Solutions demographic, clinical, analytical, imagining data at diagnosis and comorbidities during the follow-up were evaluated (Table 1).

Variables: Birthdate, age at diagnosis, gender, concomitant diseases, family history of Parkinson disease (PD), death date, severity category of disease according to Gaucher Disease Severity Score System category (GD-DS3) (mild, moderate, severe), liver size, spleen size, spleen removal, previous bone crisis and bone disease degree according to the Spanish magnetic resonance image score (S-MRI) (mild: 0–4; moderate: 5-8; severe > 9), bone mineral density (DEXA), GD biomarkers (chitotriosidase activity (ChT), CCL18/PARC and Glucosilsphyngosine (GluSph) concentrations), B12 vitamin level, iron concentration, serum ferritin, cholesterol, triglycerides, high density lipoprotein cholesterol (HDL), Low density lipoprotein cholesterol (LDL), aspartate transaminase (AST), alanine transaminase (ALT), gamma-glutamyl transferase (GGT), acid phosphatase, bilirubin, hemoglobin concentration, white blood cells (WBC) count, platelets count, serum gammaglobulin fraction, immunoglobulins (IgG-, IgA-, IgM) -serum concentrations, glucocerebrosidase (GCase) activity, *GBA* genotype (NM_000157), presence of absence of the variant NM_0003465:c.1049_1072dup24 on *CHIT1*, age to start therapy, type of therapy (enzyme replacement therapy (ERT) or substrate reduction therapy (SRT) or no therapy, new bone crisis or joint replacement, development of malignancies or PD, collected over a follow-up period of 5 to 25 years.

The aimed conditions for which the analysis sought correlations were the presence of severe bone disease at diagnosis, development of bone crisis during follow-up, and the development of neoplasia or PD.

### Statistical analysis

The statistical analysis of the data was made in two parts.

#### Baseline data analysis

A descriptive analysis was performed by splitting the variables between numerical and categorical. To establish correlation, Pearson, Chi-Square, Mann-Whitney and Mann-Whitney normalized tests were used.

#### Prediction model

Based on the results of the first step and the correlation between the different variables, we proceeded to the development of a predictive model using decision trees.

To implement the models, a training and validation cohort [30] were used, with application of the cross-validation technique [31]. This allowed us to offer an estimate of errors. The models have been built only with GD1 patients; GD3 patients have been ruled out because they can die prematurely due to the severity of their disease. Standard quality metrics such as test sample size, accuracy, sensitivity, specificity, odds ratio (OR), positive predictive value (PPV), true positives (TP), true negatives (TN), false positives (FP), false negatives, area under the receptor operator curve (AUC) were calculated. Preprocessing, data analysis and modelling were carried out through the programming language R programming language (version 3.6.2), by using, among others, the following packages: car, ggplot2, vcd, GGally, plyr, igraph, rpart, dplyr [32–34].

## Results

### General characteristics

Most patients were GD1 (337 GD1; 94. 4%) and the rest were GD3 (21, 5.6%). The most frequent *GBA* genotype was complex heterozygosity (290; 81.0%) with the most common variant being NM_000157:c.1226A > G (353/716 alleles; 49.3%). Forty-seven GD1 patients (13. 9%) were homozygous for c.1226A > G, and 9 GD3 patients (42.8%) were homozygous for c.1448 T > C. Diagnosis was made before the year 2000 for 168 (46. 9%) and 36 (10.1%) died before this study. Most of patients (193, 53. 9%) were treated with ERT. At diagnosis, 65 patients (18.2%) were splenectomized, and 139 (38.89%) had advanced bone disease with bone complications. Regarding comorbidities, 14 (4.1%) GD1 patients developed monoclonal gammopathy of undetermined significance (MGUS), another 14 (4.1%) suffered PD, and 20 (5.6%) malignant neoplasia (Table 2).

**Table 2:** General characteristics

**Characteristics**	Total: 358	100%
Mean age at diagnosis (range)	28.1 y.o. (87–0.5)	
Mean age at therapy (range)	31.5 y.o. (1-83)	
ChT activity^a^ (range)	13,604.37 (67.0–65,497.01)	
CCL18/PARC concentration^b^ (range)	590.52 (35–3895)	
GluSph concentration ^c^	34.02 (1.10–321.06)	
Serum ferritin	568. 7 (14.0–2811.0)	
S-MRI mean score (range)	11.0 (2-21)	
	N	%
***GBA*** **genotype (NM_000157.** 4**) GD1**^**d**^	337	94. 15
[c.1226A > G] + [c.1226A > G]	47	13.91
[c.1226A > G] + [c.1448 T > C]	113	33. 43
[c.1226A > G] + [other]	146	43. 19
[other] + [other]	31	9.47
***GBA*** **genotype (NM_000157.** 4**) GD3**^**d**^	21	5.85
[c.1448 T > C] + [c.1448 T > C]	9	42.86
[c.1448 T > C] + [other]	7	33.33
[other] + [other]	5	23.81
**Diagnosis**		
Index-case	276	76.88
Family study	83	23. 12
**Gender**		
Male	191	53.20
Female	168	46.80
**Severity score index (DS3)**		
Mild	213	59.33
Moderate	102	28.41
Severe	27	7.52
**Comorbidities**		
Family history of PD	42	11.69
Development of PD	17	4.73
Spleen removal	65	18.10
Bone crisis during follow-up	81	22.56
Cancer and MGUS during follow-up	34	9.47
Other comorbidities	85	23.68
Dead	37	10.31

^a^ChT activity was analyzed in 313 cases, ccases with double presence of polymorphism in the gene encoding ChT (*CHIT1;* MIM*600031) associated with a reduction in ChT activity, causing underestimation and consequent misinterpretation and have not been considered in this section

^b^CCL18/PARC concentration was analyzed in 248 patients

^c^GluSph concentration was analyzed in 77 patients

^d^*GBA* genotype according with the reference sequence NM_000157. 4, other variants meant no c.1266A > G, neither c.1448 T > C

### Correlations between numerical variables

**Fig. 1:**
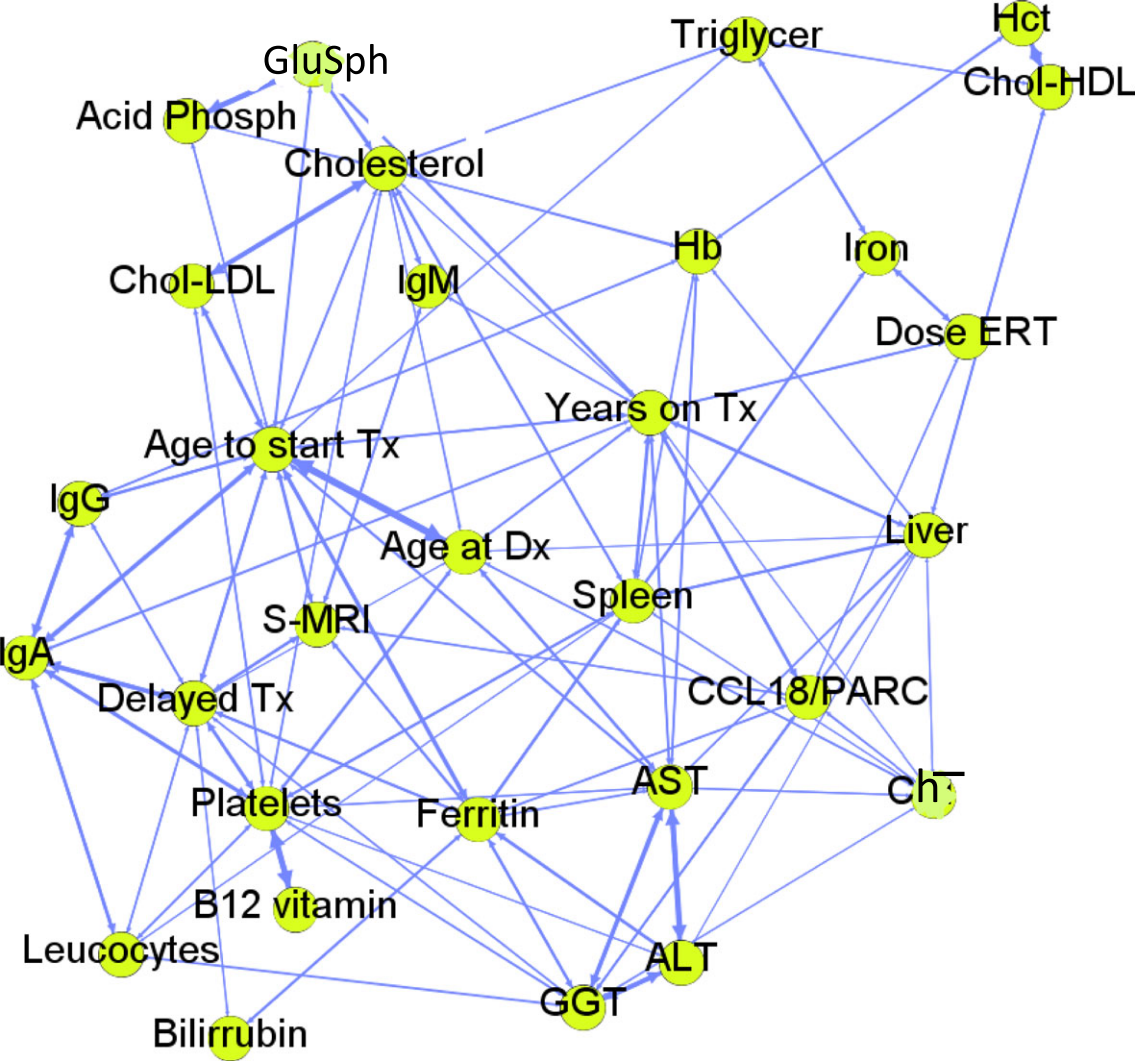
Correlation network between numerical variables. Nodes are the variables and a link is established between them if correlation is statistically significant (*p*-value ≤0.05). Those nodes that are joined by stronger links are placed closer, while those that are unrelated are further away. GluSph: Lyso-glucosylsphingosine; Triglycer: serum triglycerides; Hct: hematocrit; Acid Phosphatase; Chol-HDL: cholesterol HDL, Chol-LDL serum concentration: cholesterol LDL serum concentration; IgM: immunoglobuline M serum concentration; Tx: therapy; Delay Tx: time since diagnosis to start of therapy

A detailed correlation between the numerical variables (Table S1) and categorical variables (Table S2) can be found in supplemental material. A graph was constructed to provide a representation of how the different variables are related to each other, not only in pairs but in a global way (Fig. 1). In this graph, the nodes are the different variables and a link is established between two of them if the correlation (Pearson’s r) calculated between them is statistically significant (*p* ≤ 0.05). The weight of the link is equal to the correlation between the two variables. The position algorithm used for its creation tries to place more closely those nodes that are joined by stronger links, while those that are unrelated are further away. The highest correlation was established between the age of diagnosis and the age of onset of treatment. The statistical analysis was performed in order to stablish correlation among all variables, however, there were some correlations that need to be taken carefully in an individualized manner, in special the one involving baseline characteristics and variables such as the age at diagnosis, time since diagnosis to therapy, time on therapy. At this respect, for example, some patients did not start therapy because the ERT was not available, and as consequence their age at therapy correlates with the delay of therapy.

### Correlation between categorical variables

Table S2 from supplemental material shows the significance of the correlation between the categorical variables. There was a high correlation between spleen removal and the presence of bone disease (χ^2^_n_ = 10.87, *p* < 0.01) and repeat bone crises (χ^2^_n_= 15.93, *p* < 0.01). Almost all of the patients who suffered new bone crises had previous bone lesions (χ^2^_n_ = 30.47, *p*-value< 0.01).

Family history of PD and *GBA* genotypes no NM_000157. 4:c.1226A > G in homozygosity were the variables related to the development of PD (χ^2^_n_= 4.58, *p* < 0.01 in the correlation between having PD’s or not and the set of 11 different genotypes).

**Fig. 2:**
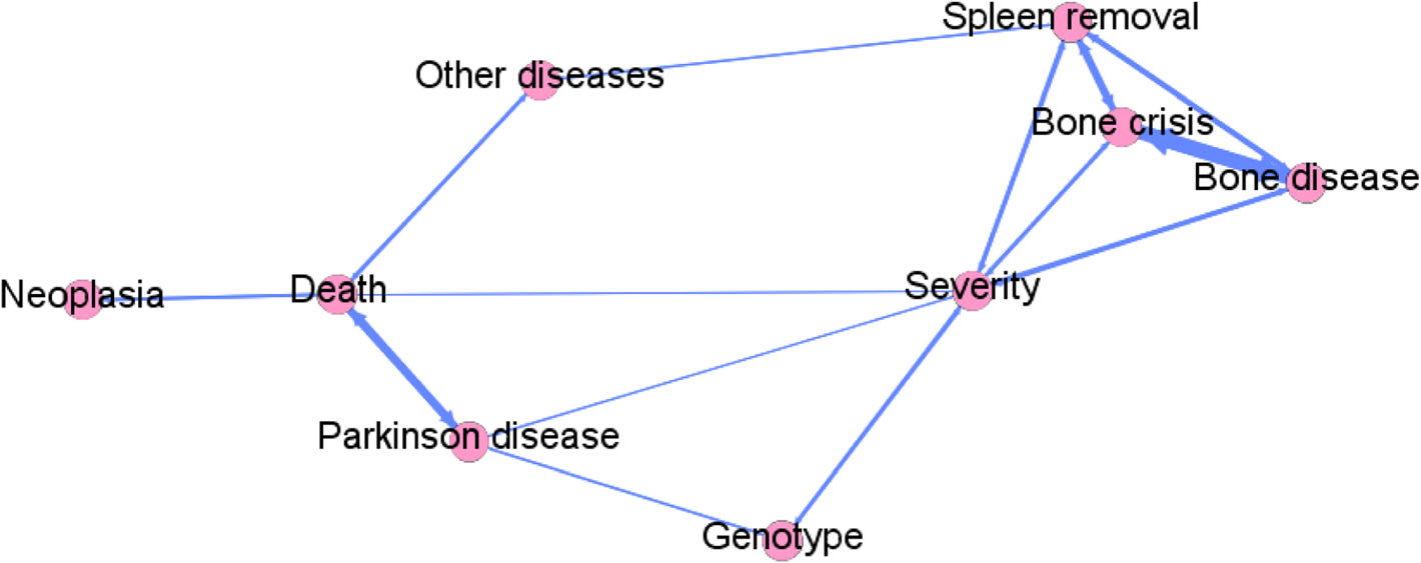
Correlation network between categorical variables, where the nodes are the different variables and a link is established between two of them if the correlation calculated between them is statistically significant (*p*-value ≤0.05). Those nodes that are joined by stronger links are placed closer, while those that are unrelated are further apart

The last correlation between categorial variables with statistical significance was cancer development (not only hematological) and spleen removal (χ^2^_n_ = 3.80, *p* = 0.05) (Fig. 2).

### Correlation between numerical variables and conditions

To stablish correlation between the presence of conditions such as severe bone disease, repeated bone crisis, Spleen removal, Parkinson Disease and neoplasia with the numerical variables the normalized Mann-Whitney test was used; for this two levels were stablished, level 1 the absence of the condition and level 2 the presence of the condition (Table S3 supplementary material).

#### Bone disease

**Fig. 3:**
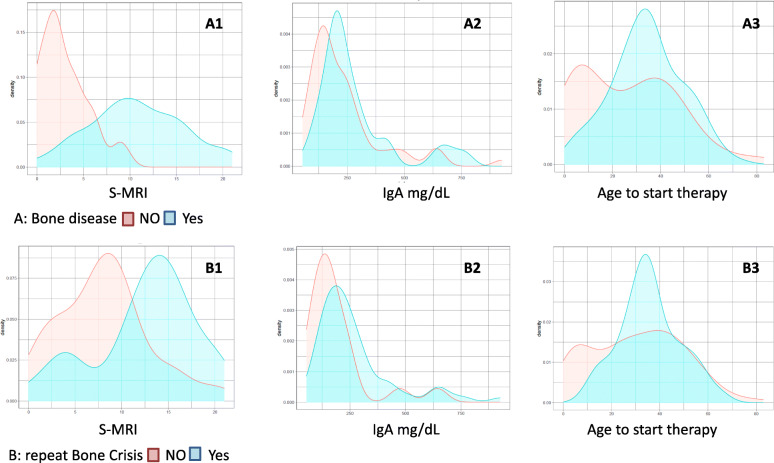
Correlation between numerical variables and bone disease. Histograms: **A** Correlation between S-MRI, IgA and age to start ERT and severe bone disease. **B** Correlation between S-MRI, IgA and age to start ERT and repeat bone crisis

The numerical variables that showed the main relevance for severe bone disease at diagnosis were the S-MRI (U_n_ = 0.98, *p* < 0.01) and IgA levels (U_n_ = 0.93, *p* = 0.01), Table S3, supplemental material. Nevertheless, there were other variables that present relatively high correlations and low *p*-values, such as high levels of ferritin (U_n_ = 0.85, *p* = 0.06), triglycerides (U_n_ = 0.75, *p* < 0.01), delayed age at diagnosis (> 9.5y.o.) (*p* < 0.001), time in years between diagnosis and the start of treatment (U_n_ = 0.67, *p* = 0.01) or delayed age of initiation of ERT (U_n_ = 0.61, *p* = 0.01) (Fig. 3A1).

The same happens with the appearance of successive bone crises during ERT. The variables that correlate and have greater significance are S-MRI (U_n_ = 0.92, *p* < 0.01), high IgA levels (U_n_ = 0.91, *p* = 0.08), and delayed age of initiation of ERT (U_n_ = 0.51, *p* = 0.07) (Fig. 3B1).

**Fig. 4:**
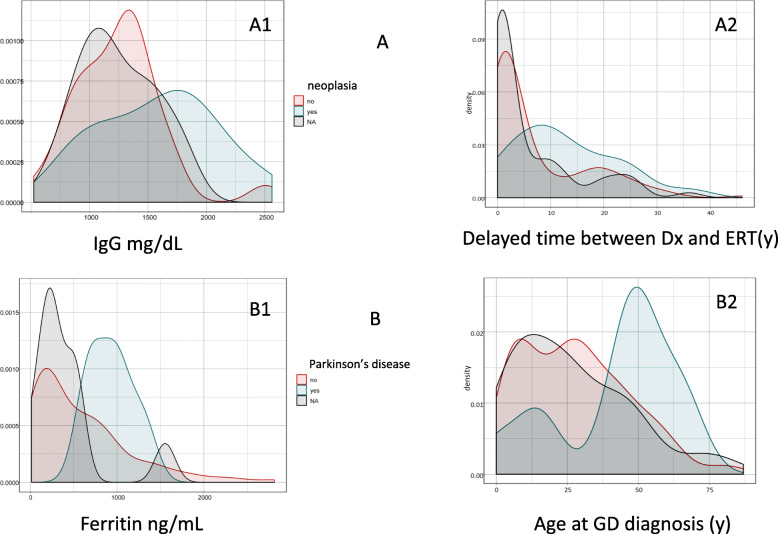
Correlation between numerical variables with the development of neoplasia or Parkinson’s disease. Histograms: **A** Correlation between increased serum IgG levels, time delay between GD diagnosis and the start of ERT with the development of neoplasia. **B** Correlation between increased serum ferritin levels, age of GD diagnosis with the development of neoplasia

The analysis considering the mean age at diagnosis minus the mean age to start therapy (U_n_ = 0.62, *p* = 0.00001) (Table S3 Supplementary material) (Fig. 4A2).

#### Neoplasia and Parkinson’s disease

High levels of IgG (U_n_ = 0.91, *p* = 0.01) and time delays before the start of therapy (mean age at diagnosis: 28.1 y.o. (0. 5-87); mean age at start therapy 31.5 y.o. (1-83); mean delay time: 7.8 years (0–46) (U_n_ = 0.70, *p* = 0.00) were related to the development of neoplasia (Fig. 4A).

In relation to the occurrence of PD, the numerical variables that have a significant correlation were elevated ferritin levels (U_n_ = 0.92, *p* = 0.04) and age at diagnosis (U_n_ = 0.45, *p* = 0.01); in this last correlation the age of PD onset probably has more weight (Fig. 4B). The significant correlations are presented in Table S3 of supplemental material.

### Correlation between categorical variables

All correlations observed between categorical variables are shown in Fig. 2.

High correlation were found between spleen removal and the severe bone disease and repeated bone crises (*p* = 0.0001). Almost all of the patients who suffered new bone crises had previous bone lesions (*p* = 0.0005) in spite of long-term ERT exposure.

The family history of PD and *GBA* genotypes other than homozygous NM_000157.4:c.1226A > G were also found to be statistically associated with PD development (*p* < 0.01).

The last correlation between categorial variables with statistical significance was cancer

development (not only hematological) and spleen removal (*p* = 0.05) Fig. 2. (Table S2 supplementary material).

### Generation of predictive models for complications by means of decision trees

**Fig. 5:**
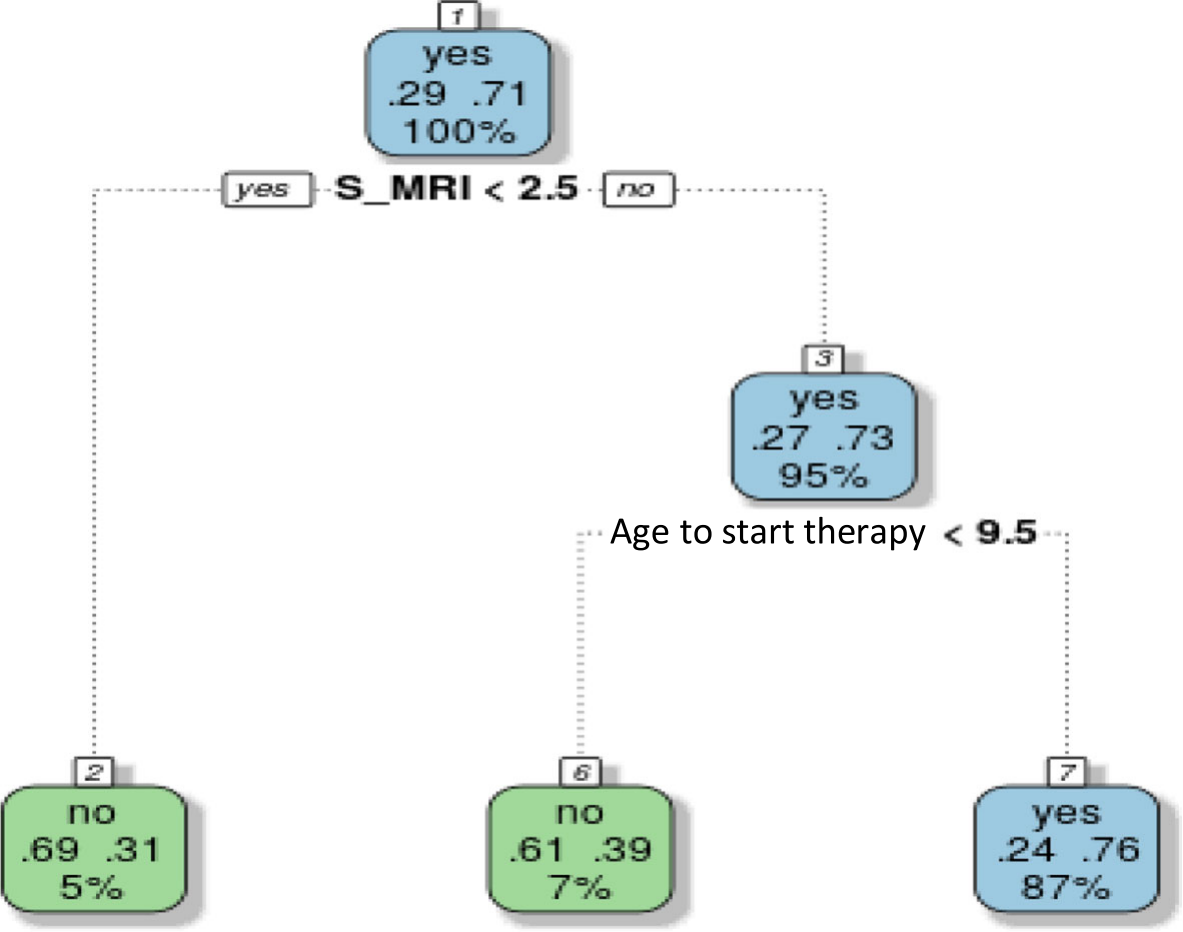
Decision tree related to the development of bone disease. The information that appears in each node includes (top down): the value of the target variable assigned by the algorithm: develops bone disease: yes/no. the ratio of patients in this node who had (left) / did not have (right) bone disease. percentage of the total population included in this node. For the prediction of bone complications, a mild bone marrow infiltration of 2.5 points by the Spanish Magnetic Resonance Imaging Score (S-MRI) with the delay of the start therapy over an age 9.5 years were the two characteristics selected by the prediction model

Decision trees show the best prediction for the development of severe bone disease in patients with an S-MRI > 2.5 who started therapy after 9.5 years; 87% of patients with these characteristics developed a severe bone disease (Fig. 5).

For neoplasia, a higher risk was found when IgG > 1725 mg/dL and age of diagnosis > 60 y.o.

In the case of PD, it was not possible to design a tree that improves the prediction of the risk of disease development, because the percentage of patients was very small. However, it has been observed that there is an important correlation with the *GBA* genotype (*p* < 0.01) and also with the existence of relatives with PD (*p* = 0.08), although this was not statistically significant. In the supplemental material, Tables S3-S4 show the statistical significance of correlation among the selected variables used for the algorithms.

## Discussion

Large-scale data (big data) in our case when referring to a rare disease have been adapted to the number of cases available; this kind of analysis is a new tool that has recently been incorporated into biomedical activity; machine learning is the study of computer algorithms that improve automatically through experience and it involves a wide series of algorithms, classification and regression models such as decision trees being some of them [26–29]. This methodology is especially useful for obtaining pooled information on the diversity of outcomes and identifying prognostic factors potentially related to disease complications [35]. In rare disease research, this is of particular interest due to the scarcity and the spread of the data among the different centers [36]. Various approaches have been applied in the area of rare diseases, especially in looking for genetic associations [37] and making correlations between genotype and phenotype [38].

Registries have an important role in this kind of analysis, because they include complete information about patients, which is especially important for rare disease research. This collected information helps in diagnosis, patient management, treatment strategy planning, health care planning and follow-up. It enables the acceleration of research and paves new pathways for personalized medicine [39, 40].

This study is the first attempt to establish a correlation network among different biochemical and clinical characteristics in a national-base cohort. We have aimed to analyse diagnostic data and to relate them with long-term complications as bone crises, development of neoplasia or PD, which are the most common and disabling complications [41–45].

Two observations, already accepted in Gaucher research, were also confirmed in this machine-learning study: first, the fact that spleen-removal patients have a higher risk of presenting more serious and extensive bone disease; second, our observation that almost all patients with new bone crisis – despite having received long-term ERT – had previous bone lesions, which remind us that the most feared complication in GD1 are not solved merely by starting ERT. These two facts confirm previous reports and provide validity of our analysis [42, 45–47]. In addition, genotypes different from homozygous NM_000175. 4:c.1226A > G are significantly correlated with bone disease (*p* = 0.05). This last observation is in line with the observation that c.1226A > G variant provides a mild phenotype [48, 49].

It is a priority to identify accurate risk factors of bone crisis to improve treatment dosage and to avoid this complication. The standard biomarkers related to GD (ChT activity, CCL18/PARC and GluSph concentrations) have been discarded as risk factors for bone complication [50, 51] even though their concentration will be increased during bone crisis, due to the acute inflammatory event [45, 49]. This reminds us of the importance to continue searching for other biomarkers. Our results confirm the lack of association between these biomarkers and disease outcomes, but other biomarkers, such as high levels of ferritin, show a tendency in patients with advanced bone disease although it was not statistically significant.

Surprisingly, the high serum IgA concentration correlates with the degree of bone involvement and with the development of bone crisis (*p* = 0.001). The age of onset of treatment (mean 30.6 y.o.) (*p* = 0.01) also shows a clearer relevance for the occurrence of bone crises (*p* = 0.01).

In this study, the development of malignancies appears strongly correlated with the delayed age at the start of ERT (*p* < 0.01) and the increased concentration of IgG (*p* = 0.01). Many aspects remain to be unraveled in the complexity of the immune system, but aging is an important factor clearly related to humoral immune dysfunction and the appearance of malignancies [52]. Polyclonal and monoclonal gammopathies in GD patients are common [53] and we observed a significant correlation between high levels of IgG and the appearance of neoplasia [54]. However, the origin of these alterations is not fully clarified, and is attributed to the chronic inflammation state; also, it is related to an increase in levels of inflammatory cytokines such as interleukins (IL-6, IL-10) that could lead to an overproduction of immunoglobulins [53, 54]. Another hypothesis could be that B lymphocytes were activated by specific type II natural killer T lymphocytes, with a T follicular helper profile, and that the clonal immunoglobulin in GD patients and in mouse models of GD was reactive against GluSph [55].

The identification of levels of IgA as a risk factor for complication was a surprising finding; it has not been previously reported that IgA levels are related to severe bone disease and the presence of repeated bone crises in GD.

Also, our data, shown an analysis of the main clinical features of GD patients at diagnosis; in accordance with previous reports [2, 6, 10–12], general characteristics such as polyclonal gammopathies, bone pain, bone vascular lesions, hypertriglyceridemia, splenomegaly and family history of parkinsonism, would be findings that can help to identify GD patients.

The SGDR only includes GD patients from Spain, thus the main limitations for the study are the absence of a larger data set. Despite this, the included data reflect the characteristics of the disease in this country. It could be interesting to validate these findings by studying other populations with a greater number of patients; however, taking into account the homogeneity of the series and the single-country origin, the data are solid.

## Conclusions

Our work confirms previous observations such as the relationship among bone disease and splenectomy; it highlights the importance of early diagnosis and therapy and identifies new risk features such as high IgA and IgG levels for long-term complications. This is first attempt in which all the baseline diagnosis data has been included in a study to perform analysis of network correlations. This open the possibility to move forward using nowadays technology; it will help us to identified features that can predict risk for complications or maybe, if more patients can be included, a better phenotype-genotype correlation.

## Supplementary information

**Additional file 1: Table S1.** (supplementary material). Correlation between numerical variables. **Table S2.** (supplementary material). Correlations between categorical variables. **Table S3.** (supplementary material): Correlations of the conditions with numerical variables. **Table S4.** (supplementary material) Correlations of the conditions with categorical variables Bone disease.

## Data Availability

The data analysed and generated during the current study belongs to the SRGD and to the FEETEG are available under request through the corresponding author.

## Abbreviations

ALT: Alanine transferase
AST: Aspartate transferase
AUC: Area under the curve
CCL18/PARC: Pulmonary and activation-regulated chemokine
ChT: Chitotriosidase activity
DEXA: Bone mineral density exam
ERT: Enzymatic Replacement Therapy
FEETEG: Spanish foundation of Gaucher disease and other lysosomal disorders
FN: False negative
FP: False positive
GCase: Glucocerebrosidase activity
GD-DS3: Gaucher Disease Severity Score System category
GD: Gaucher’s disease
GD1: Type 1 GD
GD2: Type2 GD
GD3: Type3 GD
GGT: Gamma-glutamyl transferase
GluSph: Glucosilsphyngosine
HDL: High density lipoprotein
LDL: Low density lipoprotein
LSD: Lysosomal storage disorder
MGUS: Monoclonal gammopathy undetermined significance
OD: Odd ratio
PD: Parkinson disease
PPV: Positive predictive value
S-MRI: Spanish magnetic resonance image score
SGDR: Spanish Gaucher Disease Registry
SRT: Substrate Reduction Therapy
TP: True positive
Tx: Therapy
WBC: White blood cells
