# Supplementary material for: Identification of risk features for complication in Gaucher’s disease patients: a machine learning analysis of the Spanish registry of Gaucher disease
Source: Orphanet J Rare Dis. 2020 Sep 22;15:256. doi: 10.1186/s13023-020-01520-7 (PMC7507684; doi:10.1186/s13023-020-01520-7)
Supplement: Supplementary file 1 — Additional file 1: Table S1. (supplementary material). Correlation between numerical variables. Table S2. (supplementary material). Correlations between categorical variables. Table S3. (supplementary material): Correlations of the conditions with numerical variables. Table S4. (supplementary material) Correlations of the conditions with categorical variables Bone disease. [file 13023_2020_1520_MOESM1_ESM.docx]

**Supplemental Material**

Table S1 (supplementary material). Correlation between numerical variables.

| **variable1** | **variable2** | **Correlation** | **P-value** |
| --- | --- | --- | --- |
| IgG serum | Immunoglobulines total seum | 0.92 | 0 |
| Age at Diagnosis | Age at start of therapy | 0.8 | 0 |
| Vit_B12 | Acid phosphatase | 0.74 | 0.06 |
| Vit_B12 | ALT | -0.68 | 0.13 |
| Vit_B12 | Plaquetas | -0.67 | 0.03 |
| IgA | IgTotal | 0.67 | 0 |
| GOT | AST | 0.61 | 0 |
| Lyso_Gb1 | Acid phosphatase | 0.61 | 0 |
| HDL | Hematocrit (Hto) | 0.55 | 0 |
| Vit_B12 | LDL-cholesterol | 0.55 | 0.26 |
| Colesterol | LDL-cholesterol | 0.53 | 0 |
| GPT | GGT | 0.53 | 0 |
| Vit_B12 | GGT | -0.51 | 0.38 |
| IgA | Time since diagnosis to therapy (Delay Tx, years) | 0.51 | 0 |
| Vit_B12 | Enzymatic activity | -0.47 | 0.23 |
| GOT | GGT | 0.47 | 0 |
| IgG | IgA | 0.47 | 0 |
| Vit_B12 | Total serum billirrubin | 0.46 | 0.35 |
| IgA | Age at start of therapy | 0.46 | 0 |
| Vit_B12 | IgM | -0.42 | 0.29 |
| Plaquetas | IgA | 0.42 | 0 |
| Vit_B12 | Immunoglobulines total seum | -0.42 | 0.3 |
| Ferritina | Age at start of therapy | 0.42 | 0 |

Table S2 (supplementary material). Correlations between categorical variables

| **variable1** | **variable2** | **Χ^2^** | **p-value** | **degrees of freedom** | **Χ^2^_n_** |
| --- | --- | --- | --- | --- | --- |
| GD type | GD-SD3 | 87.36 | 0 | 2 | 43.68 |
| Bone disease | Repetead BC | 30.47 | 0 | 1 | 30.47 |
| Parkinson disease | Dead | 20.65 | 0 | 1 | 20.65 |
| GD type | Parkinson disease | 17.5 | 0 | 1 | 17.5 |
| Repetead BC | Spleen removal | 15.93 | 0 | 1 | 15.93 |
| SD3 | Parkinson disease | 28.29 | 0 | 2 | 14.14 |
| GD type | Genotype | 182.57 | 0 | 13 | 14.04 |
| Bone disease | Spleen removal | 10.87 | 0 | 1 | 10.87 |
| Other comorbidity | Dead | 10.05 | 0 | 1 | 10.05 |
| SD3 | Bone disease | 18.32 | 0 | 2 | 9.16 |
| SD3 | Spleen removal | 14.75 | 0 | 2 | 7.38 |
| SD3 | Dead | 14.42 | 0 | 2 | 7.21 |
| SD3 | Repetead BC | 12.96 | 0 | 2 | 6.48 |
| Spleen removal | Other comorbidity | 5.53 | 0.02 | 1 | 5.53 |
| Neoplasia | Dead | 5.37 | 0.02 | 1 | 5.37 |
| Genotype | GD-SD3 | 133.49 | 0 | 26 | 5.13 |
| Spleen removal | Dead | 4.99 | 0.03 | 1 | 4.99 |
| Genotype | Parkinson disease | 44.37 | 0 | 11 | 4.03 |
| Neoplasia | Spleen removal | 3.8 | 0.05 | 1 | 3.8 |
| Gender | Bone disease | 3.79 | 0.05 | 1 | 3.79 |

Table S3(supplementary material): Correlations of the conditions with numerical variables

- Bone disease

| **variable** | **level1** | **level2** | **Mann_Whitney** | **p_value** | **Mann_Whitney_Normalised** |
| --- | --- | --- | --- | --- | --- |
| S_MRI | no | yes | 110.5 | 0 | 0.98 |
| IgA | no | yes | 412.5 | 0.01 | 0.93 |
| Ferritin | no | yes | 931 | 0.06 | 0.85 |
| Triglycer | no | yes | 1582.5 | 0 | 0.75 |
| Time Dx-Tx | no | yes | 2070 | 0 | 0.67 |
| GBA act | no | yes | 2273.5 | 0.06 | 0.63 |
| Age to start ERT | no | yes | 2406.5 | 0.01 | 0.61 |
| Total Bilirrub | no | yes | 2674.5 | 0.06 | 0.57 |

- Repeated bone crisis under ERT

| **variable** | **level1** | **level2** | **Mann_Whitney** | **p_value** | **Mann_Whitney_Normalised** |
| --- | --- | --- | --- | --- | --- |
| S_MRI | no | yes | 574.5 | 0 | 0.92 |
| IgA | no | yes | 607 | 0.08 | 0.91 |
| Triglycer | no | yes | 2177.5 | 0.02 | 0.69 |
| Time Dx-Tx | no | yes | 2693 | 0 | 0.62 |
| Age to start ERT | no | yes | 3479.5 | 0.07 | 0.51 |
| AST | no | yes | 3734.5 | 0.06 | 0.47 |
| Time on Tx | no | yes | 4057 | 0 | 0.43 |
| Liver_cm | no | yes | 4436.5 | 0.08 | 0.37 |

- Spleen removal

| **variable** | **level1** | **level2** | **Mann_Whitney** | **p_value** | **Mann_Whitney_Normalised** |
| --- | --- | --- | --- | --- | --- |
| IgA | no | yes | 278 | 0 | 0.96 |
| LDL-Chol | no | yes | 438 | 0.01 | 0.94 |
| IgG | no | yes | 441 | 0 | 0.94 |
| S_MRI | no | yes | 547.5 | 0 | 0.92 |
| Ferritin | no | yes | 999.5 | 0 | 0.86 |
| IgM | no | yes | 1097.5 | 0.03 | 0.85 |
| WBC | no | yes | 1441.5 | 0 | 0.8 |
| Time Dx-Tx | no | yes | 1483 | 0 | 0.79 |
| GGT | no | yes | 1547.5 | 0 | 0.78 |
| Platelets | no | yes | 2004 | 0 | 0.72 |
| Cholesterol | no | yes | 2331.5 | 0.01 | 0.68 |
| Age to start ERT | no | yes | 2494.5 | 0 | 0.65 |
| CCL18/PARC | no | yes | 2602.5 | 0.01 | 0.64 |
| ALT | no | yes | 2628.5 | 0 | 0.63 |
| AST | no | yes | 3182.5 | 0.01 | 0.56 |
| Liver_cm | no | yes | 3869.5 | 0 | 0.46 |
| Spleen_cm | no | yes | 4096 | 0.06 | 0.43 |

- Neoplasia

| **variable** | **level1** | **level2** | **Mann_Whitney** | **p_value** | **Mann_Whitney_Normalised** |
| --- | --- | --- | --- | --- | --- |
| IgG | no | yes | 240 | 0.01 | 0.91 |
| Time Dx-Tx | no | yes | 794 | 0 | 0.7 |
| Age to start ERT | no | yes | 922 | 0.02 | 0.66 |

- Parkinson’s disease

| **variable** | **level1** | **level2** | **Mann_Whitney** | **p_value** | **Mann_Whitney_Normalised** |
| --- | --- | --- | --- | --- | --- |
| Ferritin | no | yes | 127.5 | 0.04 | 0.92 |
| HDL-Chol | no | yes | 300 | 0.04 | 0.82 |
| Triglycer | no | yes | 360 | 0.03 | 0.78 |
| Time Dx | no | yes | 893 | 0.01 | 0.45 |

To stablish correlation between the presence of conditions such as severe bone disease, repeated bone crisis, Spleen removal, Parkinson Disease and neoplasia with the numerical variables the normalized Mann-Whitney test was used; for this two levels were stablished, level 1 the absence of the condition and level 2 the presence of the condition.

Table S4. (supplementary material) Correlations of the conditions with categorical variables

Bone disease

| **variable** | **χ^2^** | **p_value** | **df** | **χ^2^_n_** |
| --- | --- | --- | --- | --- |
| Repetead BC | 31.77 | 0 | 1 | 31.77 |
| Spleen removal | 11.05 | 0 | 1 | 11.05 |
| GD-SD3 | 19.73 | 0 | 2 | 9.87 |
| Gender | 3.36 | 0.07 | 1 | 3.36 |
| Genotype | 14.05 | 0.05 | 7 | 2.01 |

Repeated bone crisis under ERT

| **variable** | **χ^2^** | **p_value** | **df** | **χ^2^_n_** |
| --- | --- | --- | --- | --- |
| None comorbidity | 31.77 | 0 | 1 | 31.77 |
| Spleen removal | 14.52 | 0 | 1 | 14.52 |
| GD-SD3 | 16.13 | 0 | 2 | 8.06 |

Spleen removal

| **variable** | **χ^2^** | **p_value** | **df** | **χ^2^_n_** |
| --- | --- | --- | --- | --- |
| Repetead BC | 14.52 | 0 | 1 | 14.52 |
| Bone disease | 11.05 | 0 | 1 | 11.05 |
| GD-SD3 | 18.23 | 0 | 2 | 9.11 |
| Other comorbidity | 4.39 | 0.04 | 1 | 4.39 |
| Neoplasia | 3.79 | 0.05 | 1 | 3.79 |

Neoplasia

| **variable** | **χ^2^** | **p_value** | **df** | **χ^2^_n_** |
| --- | --- | --- | --- | --- |
| Dead | 6.89 | 0.01 | 1 | 6.89 |
| Spleen removal | 3.79 | 0.05 | 1 | 3.79 |

Parkinson disease

| **variable** | **χ^2^** | **p_value** | **df** | **Xiχ^2^_n_2_over_df** |
| --- | --- | --- | --- | --- |
| Dead | 17.25 | 0 | 1 | 17.25 |
| Genotype | 27.47 | 0 | 6 | 4.58 |
| GD-SD3 | 7.53 | 0.02 | 2 | 3.76 |
| Parkinson in relatives | 3.16 | 0.08 | 1 | 3.16 |

Other comorbidities

| **variable** | **χ^2^** | **p_value** | **df** | **iχ^2^_n_2** |
| --- | --- | --- | --- | --- |
| Dead | 7.48 | 0.01 | 1 | 7.48 |
| Spleen removal | 4.39 | 0.04 | 1 | 4.39 |
| Gender | 3.64 | 0.06 | 1 | 3.64 |
